# Human Brain Organoids to Decode Mechanisms of Microcephaly

**DOI:** 10.3389/fncel.2020.00115

**Published:** 2020-05-08

**Authors:** Elke Gabriel, Anand Ramani, Nazlican Altinisik, Jay Gopalakrishnan

**Affiliations:** Laboratory for Centrosome and Cytoskeleton Biology, Institute für Humangenetik, Universitätsklinikum Düsseldorf, Heinrich-Heine-Universität, Düsseldorf, Germany

**Keywords:** centrosomes, primary cilia, neural progenitor cells (NPCs), induced pluripotent stem cells (iPSCs), human brain organoids, microcephaly, neurodevelopmental disorders, neurogenesis

## Abstract

Brain organoids are stem cell-based self-assembling 3D structures that recapitulate early events of human brain development. Recent improvements with patient-specific 3D brain organoids have begun to elucidate unprecedented details of the defective mechanisms that cause neurodevelopmental disorders of congenital and acquired microcephaly. In particular, brain organoids derived from primary microcephaly patients have uncovered mechanisms that deregulate neural stem cell proliferation, maintenance, and differentiation. Not only did brain organoids reveal unknown aspects of neurogenesis but also have illuminated surprising roles of cellular structures of centrosomes and primary cilia in regulating neurogenesis during brain development. Here, we discuss how brain organoids have started contributing to decoding the complexities of microcephaly, which are unlikely to be identified in the existing non-human models. Finally, we discuss the yet unresolved questions and challenges that can be addressed with the use of brain organoids as *in vitro* models of neurodevelopmental disorders.

## Introduction

Our knowledge of the mechanisms of human brain development is limited mainly because the human brain is enormously complex in its cell diversity, composition, and architecture (Northcutt and Kaas, 1995; Borrell and Gotz, 2014). Cortical expansion of the human brain is one of the most remarkable evolutionary processes of brain development that is correlated to sophisticated tasks of decision making, emotional, cognitive, and social interactions (Rilling, 2014; Reardon et al., 2018). A highly orchestrated process of neural stem cell maintenance, proliferation, migration, and interactions ensure the accurate and structurally normal cortical expansion. Perturbations in any of these individual steps can lead to neurodevelopmental disorders. Primary microcephaly is one such neurodevelopmental disorder in which brain size is markedly reduced (Jayaraman et al., 2018; Pirozzi et al., 2018).

In mammalians, brain development begins with the massive expansion of the neuroepithelium that generates radial glial stem cells (Borrell and Gotz, 2014; Florio and Huttner, 2014; Florio et al., 2017). Notably, in the human brain, the progenitor zones around the ventricular zone (VZ) are organized extensively. The sub-ventricular region consists of the inner sub-ventricular zone and the outer sub-ventricular zone, separated by an inner fiber layer. The outer sub-ventricular zone constitutes intermediate progenitors and outer radial glia. This compartmentalization, along with increased heterogeneity of neural precursor populations and their dynamic proliferative characteristics (cell cycle length, mode of division, etc.) collectively underlay the massive expansion of neural stem cells. This could lead to the highest neuron number inducing gyrification and an increase in brain size in humans. Strikingly, rodent brains, which are lissencephalic and lack the inner fiber layer, the outer sub-ventricular zone, and exhibit different dynamics of proliferation and neurogenic period (Zecevic et al., 2005; Cox et al., 2006; Molnar and Clowry, 2012). Thus, when the pathogenesis of microcephaly has been studied in mouse models, they failed to recapitulate the severely reduced brain size seen in human patients. As a result, it has been challenging to study microcephaly in model systems that do not possess the complexity of the human brain. This has been a significant limiting factor for decades and has been a challenge for developmental biologists to model microcephaly since animal models do not mirror the complex embryonic neurodevelopmental disorders occurring in humans.

With recent technological advances identifying the molecular causes of microcephaly, the interplay between genes, cellular structures, and most importantly the recent emergence of powerful 3D *in vitro* brain organoid systems have fortuitously helped to understand the mechanisms of microcephaly and underpinned the fundamental mechanisms of healthy brain development (Mariani et al., 2012; Lancaster et al., 2013; Gabriel et al., 2016; Gopalakrishnan, 2019; Setia and Muotri, 2019). In this review, we will summarize complex cellular processes in the pathogenesis of microcephaly and how the recent 3D brain organoids, also known as “brain mimetics” of the human brain have contributed to unraveling the complexities seen in microcephaly patients. Finally, we outline the critical questions that require immediate attention in the field of microcephaly research and state the current challenges that could be overcome with the use of 3D brain organoids as *in vitro* models of neurodevelopmental disorders.

## Microcephaly; Definition and Pathogenesis

The human brain constitutes approximately 2% of the total body mass, also consuming up to 20% of the total energy indicating its vitality for the organism’s survival. Deregulation of genes and pathways that have co-evolved with the human brain evolution could result in a small brain, in particular, a smaller frontal cortex, and is clinically termed as primary microcephaly. Microcephaly is classified as primary and secondary microcephaly. Primary microcephaly is a condition where abnormalities occur at the early onset of brain development resulting in an un-proportional cortical thickness. Secondary microcephaly, on the other hand, develops postnatal during infancy (Basel-Vanagaite and Dobyns, 2010; Alcantara and O’Driscoll, 2014). The term MCPH (autosomal recessive primary microcephaly) has been frequently used in clinical diagnostics of microcephaly. These two categories have been further categorized based on their symptoms. For instance, a microcephaly disorder exhibiting only reduced head circumference with mental retardation belongs to the non-syndromic type; whereas, microcephaly disorder associated with various neurological and cognitive defects falls under the syndromic type of the disease. Furthermore, the source of primary and secondary microcephaly could also be due to environmental cues as well as viral influence in addition to the well-known genetic causes. Hence they are also called acquired microcephaly. Emerging genetic mutations have further defined another class of syndromic microcephaly, which included malformations of cortices along with whole-body growth shunt, which usually is a clinical feature observed in Seckel syndrome and Microcephalic Osteodysplastic Primordial Dwarfism (MOPD) (Rauch et al., 2008; Pirozzi et al., 2018; Jayaraman et al., 2018). In contrast to Seckel syndrome, MCPH only exhibits retarded brain size (Jayaraman et al., 2018; Pirozzi et al., 2018).

Although several confusing terms and increasing branches of growth-retarded syndromes are emerging, what is undoubtedly intersecting in these disorders is microcephaly. The most frequent abnormality in microcephaly identified by the MRI imaging is diffused cortical gyral pattern where cortical layers are thin and not well layered as seen in the healthy brain (Basel-Vanagaite and Dobyns, 2010). This unambiguously points out the fact that there must be a unifying mechanism that operates in these disorders. Perhaps a tightly coordinated mechanism exists that is critical to maintaining the expanding pool of neural stem cells at the early events of brain development. Alternatively, mutations of genes with different functions, or viral infection leading to intracellular events could be distinct from those activated in genetic forms of microcephaly. However, the disease-relevant cell types of region-specific NPCs could be more prone to undergo depletion or damage under different types of stress. Overall, the depletion of this actively proliferating neural stem cell pool at the early stage of brain development could broadly affect the final mass and function of the human brain.

Congenital microcephaly is mostly caused by autosomal recessive mutations in several genes that regulate centrosome and cilia assembly, which are cellular structures that govern fundamental pathways of microtubule organization, cell proliferation, polarity, migration and cell signaling (Table 1). Indeed, the earliest identified microcephaly associated genes were implicated in centrosome biogenesis, and spindle assembly, which include molecules such as CDK5RAP2, CPAP, Cep135, Cep152, PCNT, and MCPH1 where the mutations in these genes were identified in consanguineous populations inherited via an autosomal recessive fashion (Bond et al., 2002; Bond et al., 2005; Rauch et al., 2008; Guernsey et al., 2010; Hussain et al., 2012). As mentioned before, the human neocortex differs from rodents and non-human primates in terms of neuronal numbers, which is an indicator of a positive selection in humans (Bond et al., 2005). This morphological feature suggests a sophisticated regulation of precursor cell numbers and their proliferative/differentiative ratio during neurogenesis. As an example, centrosomal proteins mutations resulting in microcephaly in humans harbor other regions besides conserved domains. Such alterations in amino acid sequences and protein expression levels appear to occur in humans specifically. This allows us to speculate their specific roles in controlling cortical expansion and neuronal number in the humans. Consequently, it is plausible that the interaction partners and biochemical pathways of these microcephaly proteins in humans could have simultaneously co-evolved (Evans et al., 2004; Hill and Walsh, 2005; Ali and Meier, 2008).

**TABLE 1:** Genes frequently mutated in primary microcephaly that plays roles in cell cycle regulation, centrosome/cilium formation, spindle orientation, microtubule organization and impaired DNA damage.

Genes	Syndrome	Subcellular localization	Modeled in patient specific brain organoids	Mechanisms revealed	References
MCPH1	Congenital microcephaly	Nucleus	No	Premature NPCs differentiation, premature chromosome condensation	Jackson et al., 2002; Passemard et al., 2011; Zhou et al., 2013; Farooq et al., 2016
ASPM	Congenital microcephaly	Centrosomes	Yes	Decreased NPCs proliferation, Less neuronal activity, cell death	Pulvers et al., 2010; Fujimori et al., 2014
WDR62	Congenital microcephaly, cortical abnormalities	Centrosomes	No	Decreased NPCs proliferation, premature NPCs differentiation	Nicholas et al., 2010; Shohayeb et al., 2019; Zhang et al., 2019
CDK5RAP2	Congenital microcephaly	Centrosomes	Yes	Decreased NPCs proliferation, premature NPCs differentiation	Bond et al., 2005; Barrera et al., 2010; Buchman et al., 2010; Babrowski et al., 2013
CENPJ/CPAP	Congenital microcephaly, Seckel syndrome	Centrosomes	Yes	Decreased NPCs proliferation premature NPCs differentiation	Al-Dosari et al., 2010; McIntyre et al., 2012; Alcantara and O’Driscoll, 2014; Aldape et al., 2019
SAS6	Congenital microcephaly	Centrosomes	No	Decreased NPCs proliferation	Khan et al., 2014; Tang et al., 2016
STIL	Congenital microcephaly	Centrosomes	No	Neural tube defects	Consortium et al., 2009; Amartely et al., 2014
CEP152	Congenital microcephaly, Seckel syndrome	Centrosomes	No	Decreased NPCs proliferation	Guernsey et al., 2010; Kalay et al., 2011
CEP63	Seckel syndrome	Centrosomes	No	Increased neuronal death, increased mitotic error	Alcantara and O’Driscoll, 2014; Marjanovic et al., 2015
NDE1	Congenital microcephaly,	Centrosomes and spindle microtubules	No	Decreased NPCs proliferation	Alcantara and O’Driscoll, 2014; Baffet et al., 2016
PCNT	Congenital microcephaly, Seckel syndrome, MOPD type II	Centrosomes	No	Decreased NPCs proliferation, aberrant mitosis, missegregation of chromosomes	Griffith et al., 2008; Miyoshi et al., 2009; Benmerah et al., 2015
RTTN	Congenital microcephaly, dwarfism, cerebellar abnormalities	Centrosomes	No	Abnormal spindles, centriole structures	Shamseldin et al., 2015; Chen et al., 2017
KIF5C	Cortical dysplasia	Spindles	No	Abnormal microtubule function	Poirier et al., 2013
KIF2A	Cortical dysplasia	Spindles	No	Abnormal axon branching, abnormal microtubule function	Poirier et al., 2013
KIF11	Congenital microcephaly	Centrosomes, spindle, and cilia	No	Abnormal spindles and reduced NPCs proliferation	Ostergaard et al., 2012
KIF14	Congenital microcephaly, Meckel syndrome	Centrosomes, spindle	No	Increased neuronal cell death, abnormal cell migration	Moawia et al., 2017
TUBA1A	Cortical abnormalities, tubulinopathy	Variable, microtubule	No	Abnormal neuronal migration	Wei et al., 2019
TUBG1	Cortical abnormalities, tubulinopathy	Variable, microtubule	No	Abnormal neuronal migration	Poirier et al., 2013
TUBB2B	Cortical abnormalities, tubulinopathy	Variable, microtubule	No	Abnormal neuronal migration	Romaniello et al., 2012
CEP135	Congenital microcephaly	Centrosomes	No	Abnormal centriole structures, disorganized spindles, reduced NPCs proliferation	Hussain et al., 2012; Lin et al., 2013
CDK6	Congenital microcephaly	Centrosomes	No	Abnormal spindle, unknown mechanisms	Hussain et al., 2013
CIT	Congenital microcephaly, dwarfism	Mid body	No	Mitotic delay, impaired cytokinesis, multipolar spindles, genomic instability, cell death	Li et al., 2016; Shaheen et al., 2016
Ninein	Seckel syndrome	Centrosomes	No	Defective migration, neuroectoderm defects	Dauber et al., 2012
NBS1	Congenital microcephaly, Nijmegen breakage syndrome	Nucleus	No	Double strand break repair deficiency	Varon et al., 1998
ATR	Seckel syndrome	Nucleus	No	Mitotic delay, impaired cytokinesis, double strand break repair deficiency	O’Driscoll et al., 2003
XLF/Cernunos	Congenital microcephaly	Nucleus	No	Double strand break repair deficiency	Buck et al., 2006
XRCC2	Congenital microcephaly	Nucleus	No	Double strand break repair deficiency neuronal death	Deans et al., 2000
XRCC4	Congenital microcephaly	Nucleus	No	Double strand break repair deficiency neuronal death	Gao et al., 1998
Ligase IV deficiency	Congenital microcephaly	Nucleus variable	No	Double strand break repair deficiency	Barnes et al., 1998
XPA-XPG	Xeroderma Pigmentosum, Microcephaly, Variable	Nucleus variable	No	Double strand break repair deficiency	Anttinen et al., 2008
ERCC6, ERCC8	Cockayne Syndrome microcephaly	Nucleus variable	No	Nucleotide excision repair and base excision repair deficiency	Jackson et al., 2002; Lin et al., 2005
TTDA	Congenital microcephaly	Nucleus variable	No	Double strand break repair deficiency	Chu and Mayne, 1996; Faghri et al., 2008
DNAPK	Congenital microcephaly, Seizures, Neuronal death	Nucleus variable	No	Double strand break repair deficiency	Vemuri et al., 2001

Apart from being a centrosome-linked syndrome, microcephaly is also caused by mutations occurring in DNA repair proteins (Jayaraman et al., 2018). In many human DNA repair defects, the repair engine counteracting DNA damage caused exogenously (i.e., radiation and toxic substances) or endogenously (i.e., base mismatch, strand breaks, stalled replication, high amount of reactive oxygen species, etc.) becomes dysfunctional (Table 1). Considering the rapid proliferative capacity of NPCs in the VZ and high level of inherent oxidative DNA damage during embryogenesis, a strictly orchestrated DNA repair pathway must be rendered for the maintenance of these stem cells for healthy brain development. When this sophisticated system fails, genomic instability occurs, which in turn could trigger NPCs differentiation or cell death (Yoshihara et al., 2017; Henry et al., 2018; Su et al., 2019). Though fate is determined according to the type of repair pathway activated, these aberrations, in each case, could potentially cause microcephaly (Ruzankina et al., 2007; Orii et al., 2006). In this context, patient-specific human brain organoids possess a great potential to decode a broad range of cellular defects occurring during human brain development. Although several DNA repair mutations are attributed to congenital microcephaly, to date, none of the DNA damage-related diseases have been modeled using brain organoids.

Besides the rare incidences of inherited microcephaly, the recent Zika virus (ZIKV) pandemic in the Americas has received significant attention due to its notorious nature of causing microcephaly (Cugola et al., 2016; Qian et al., 2016; Ventura et al., 2016; Gabriel et al., 2017; Wolf et al., 2017). This has further highlighted the vulnerability of the human brain for any developmental defects mediated by viral infections. Nonetheless, the mechanisms underlying the adverse neurodevelopmental abnormalities witnessed in both inherited microcephaly and acquired microcephaly are still mostly unknown. In addition, clinical studies or experiments with model systems that are distantly related to disease relevancy provide insufficient insights for understanding how and why neural stem cells are depleted in the developing human brain.

## What Are 3D Brain Organoids, and Why to Use Them Now?

**FIGURE 1:**
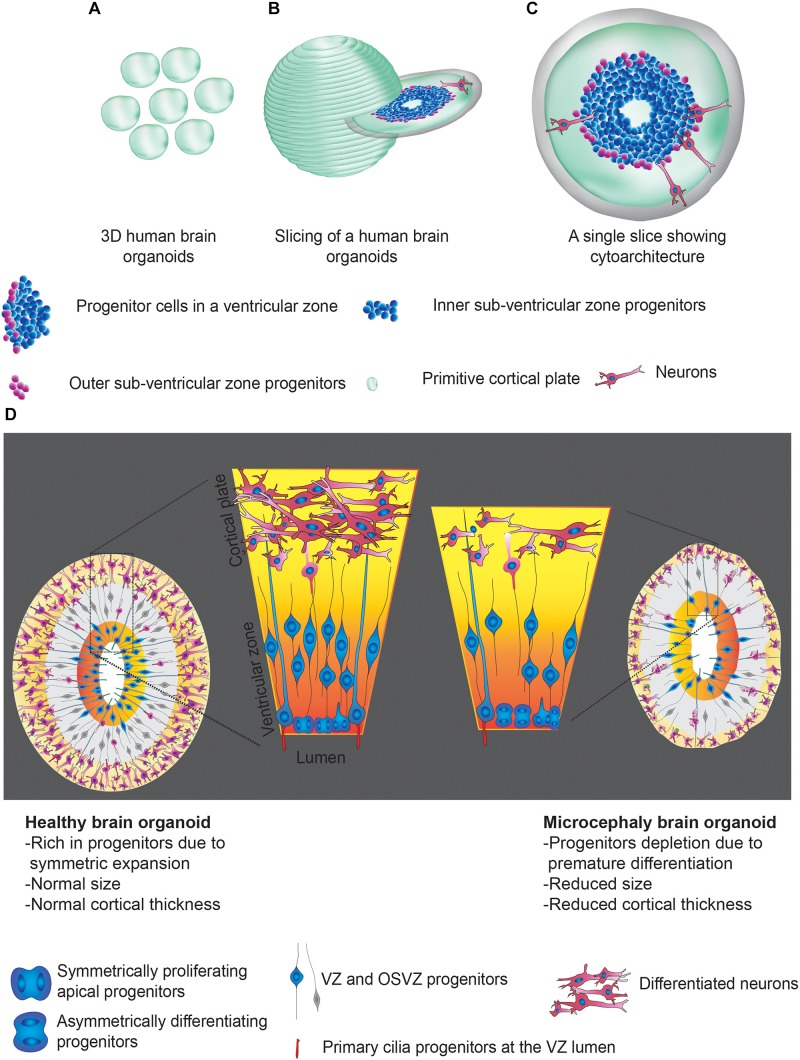
Human brain organoids and their use in modeling the mechanisms of microcephaly. **(A)** Cartoonist representation of 3D human brain organoids. **(A)** Group of brain organoids. **(B)** Slicing off a 3D organoid. **(C)** An exemplary slice showing apicobasal progenitors in a ventricular zone Legends for the specific region or cell types are given. **(D)** Schematics explain possible structural abnormalities that could occur between healthy (left) and microcephaly (right) brain organoids. Microcephaly can be caused by genetic mutations (inherited microcephaly) or ZIKV infections (acquired microcephaly). In both cases, what appears to be shared is premature differentiation of NPCs leading to cortical thinning and overall size reduction. Note that control organoid displays NPCs whose division plane is mostly horizontally oriented to the lumen of the ventricular zone, a signature of symmetric expansion. In microcephaly organoids, the division planes of NPCs are mostly vertical. Legends for the specific region or cell types are given. These figure adapted from Gabriel et al. (2017).

3D brain organoids are self-organized structures derived from human pluripotent stem cells, which have helped to understand several aspects of brain development (Giandomenico and Lancaster, 2017; Gopalakrishnan, 2019; Setia and Muotri, 2019). Importantly, these 3D structures display tissue-like morphologies containing polarized radial glia, intermediate progenitors, and layer-specific cortical neurons recapitulating several aspects of the developing human brain (Mariani et al., 2012, 2015; Kadoshima et al., 2013; Lancaster et al., 2013; Pasca et al., 2015). Organoid culturing methods, which excluded inductive signals, have led to the generation of whole-brain organoids with a primitive cortical plate, including regions mirroring forebrain, hindbrain and midbrain (Lancaster et al., 2013; Gabriel et al., 2016). Strikingly, these 3D structures constituted specific cell types that were spatially restricted apicobasally similar to VZ of the mammalian brain (Figure 1). Thus, 3D brain organoids uniquely serve as alternative model systems to address challenging questions in understanding the pathomechanisms of microcephaly using 2D cell culture and rodent models. The uniqueness of brain organoids is substantiated by the growing number of evidence that further urge the use of 3D brain organoids to model human microcephaly. We summarize some of them here as below.

Modeling microcephaly in mice often required the complete ablation of a gene implicated in the assembly of centrosomes, cilia, spindle apparatus, or DNA repair mechanisms (Wang et al., 2009; Barrera et al., 2010; Buchman et al., 2010; Lizarraga et al., 2010; Alkuraya et al., 2011; McIntyre et al., 2012; Insolera et al., 2014). Such a strong perturbation has been unusually observed in inherited microcephaly in human patients. To date, most of the inherited gene mutations causing microcephaly in human patients included point mutations, single amino acid substitutions, or truncations. In most cases, these mild perturbations led to the generation of at least partially functioning proteins, which still were not sufficient to rescue microcephaly phenotypes. Patient mutations in mouse models, in contrast to knockout models, showed only mild microcephaly phenotypes and didn’t give insights into the mechanism at the cellular level. This contrasting difference between the brain phenotypes implies that the human brain is susceptible even when the gene is mildly perturbed.

Moreover, there is yet no evidence that a disease-causing mutation in the human brain could result in the analogous microcephalic brain in mouse models. As examples, CDK5RAP2 or ASPM mutant mice did not exhibit a severely reduced brain size, as was observed in human patients (Barrera et al., 2010; Buchman et al., 2010; Lizarraga et al., 2010; Pulvers et al., 2010). Likewise, Nde1-deficient mice did not display microcephaly phenotypes as, seen in human patients (Alkuraya et al., 2011). In this line, complete ablation of CPAP/Sas-4 was required to view evident microcephaly phenotypes in the mouse brain, suggesting that mouse neural progenitor cells (NPCs) are not as susceptible as human cells (Insolera et al., 2014).

The evidence collected from *in vivo* studies allows us to speculate the presence of a crucial functional difference in brain evolution, explaining why the human brain is much more sensitive and vulnerable than that of rodents. It is possible that mouse NPCs do not extensively proliferate before the onset of neural differentiation. In other words, in the mouse brain, NPCs proliferation and differentiation are not two distinct processes, meaning that there could be an existing steady state of NPCs differentiation, which goes hand in hand with its proliferation. On the other hand, it is likely that in human brain development, NPCs first extensively proliferate to accomplish a sufficient pool of symmetrically expanding NPCs before the onset of neural differentiation, suggesting that in humans, NPCs proliferation and differentiation are seemingly two distinct processes. Verifying this hypothesis in the developing human brain is an arduous task (Rakic, 1995; Huttner and Kosodo, 2005; Geschwind and Rakic, 2013; Florio and Huttner, 2014).

Indeed, 3D brain organoids mirroring early events of human brain development have convincingly helped to verify this aspect. Analyzing the division planes of radial glial cells (RGs) at the early developmental stages of brain organoids revealed that the majority of RGs division planes are horizontally oriented, a signature of symmetric expansion (Lancaster et al., 2013; Gabriel et al., 2016; Gabriel et al., 2017). This is key evidence that human RGs at the early stages of brain development are determined to symmetrically expand to accomplish a sufficiently large pool of NPCs to generate a structurally normal-sized brain. Strikingly, division planes of RGs in rodents do not seem to follow the human rule. In contrast to human RGs, rodent RGs exhibit mixed dynamics displaying both horizontal and vertical division planes suggesting symmetric expansion is simultaneously coupled to differentiation. Studying the kinetics of RGs division planes with respect to the ventricular lumen of the developing brain is a crucial aspect in understanding the process of early neurogenesis. 3D human brain organoids offer this unique opportunity of analyzing the kinetics of RGs division planes.

In summary, one should appreciate that these mouse-based studies provide valuable insights into the early events of brain developmental mechanisms; yet, they do not sufficiently shed light on the complexes processes of human microcephaly. This, in turn, necessitates the need of 3D human brain organoids as a complementary model system that reflects the microenvironment of the human brain. The surprising trend, however, is that only a limited number of microcephaly patient-specific brain organoids have been generated to study the pathomechanisms observed in human patients (discussed as below). Nevertheless, these studies have unequivocally identified a surprising mechanism that underlay the formation of small-sized brain organoids as a consequence of impaired proliferation and premature neuronal differentiation of neural progenitors (Lancaster and Knoblich, 2014; Gabriel et al., 2016).

## Mechanisms of Microcephaly Revealed by Patient-Derived Brain Organoids

So far, only three independent centrosome-related patient-specific brain organoids have been characterized, which were generated from patient-derived iPSCs carrying mutations in CDK5RAP2, CPAP and ASPM (Lancaster et al., 2013; Gabriel et al., 2016; Li et al., 2017; Zhang et al., 2019). Thus, given the vast number of genetic mutations that cause microcephaly in humans, patient-specific microcephaly organoids are remarkably understudied. One of the limiting factors is reduced viability or instability of iPSCs due to mutations affecting centrosome structures, which are critical for fundamental cellular functions. Even then, a few numbers of patient-specific organoids thus far studied have significantly enhanced our understanding of the mechanisms that are derailed in microcephaly. In the first example, Lancaster et al. have successfully generated stable iPSCs from a patient that carried a compound heterozygous nonsense mutation in CDK5RAP2 (Lancaster et al., 2013). Using patient-derived iPSCs, the authors generate brain organoids, which were significantly smaller than the control groups. Thus, the first organoid generation protocol received the most sense of it because they could generate microcephaly brain organoids and elegantly demonstrate that patient-specific organoids exhibit the phenotypes of microcephaly patient brains. CDK5RAP2 is a pericentriolar material (PCM) protein in a centrosome (Zheng et al., 2014; Ramani et al., 2018). PCM is critical for centrosomal functions as it harbors microtubule-organizing centers. Thus, it is the PCM from where spindle microtubules emanate. Mutations in CDK5RAP2 and its homologs in various model systems have resulted in aberrantly functioning centrosomes (Zheng et al., 1995; Avidor-Reiss and Gopalakrishnan, 2012). However, the consequences of aberrantly operating centrosomes in rapidly proliferating human NPCs have never been studied until Lancaster et al. have demonstrated the critical role of centrosomes in symmetrically expanding human NPCs. It is noteworthy that CDK5RAP2 mutant mice did not exhibit a severely reduced brain size, as was observed in human patients (Barrera et al., 2010; Lizarraga et al., 2010).

As a PCM component, CDK5RAP2 is recruited to a centrosome via interacting with another conserved centrosomal protein CPAP. CPAP is a centriole wall protein required to assemble and recruit PCM proteins to a developing centrosome (Gopalakrishnan et al., 2011; Jingyan and Glover, 2012; Lawo et al., 2012). Loss of function CPAP mutants was embryonic lethal in a variety of model organisms except in flies where the mutant flies could ultimately develop (Basto et al., 2006). Despite successfully developing, these mutant flies were uncoordinated due to the defects in ciliary functions. To date, several independent CPAP mutations have been sequenced in microcephaly patients whose mechanisms underlying microcephaly remained unknown. Besides, whether primary cilium plays a role in microcephaly pathogenesis has never been tested. In the second example, Gabriel et al. generated stable iPSCs from Seckel syndrome patient-derived fibroblasts, which harbored a splice-site mutation in CPAP, resulting in homozygous G-C transition in the last nucleotide of intron 11. This perturbation resulted in the deletion of exons 11, 12, and 13 (Al-Dosari et al., 2010). Brain organoids generated using equivalent starting numbers of iPSCs revealed that patient-specific brain organoids were significantly smaller than that of the control groups again, demonstrating that brain organoid is versatile models to exhibit progenitor biology-related defects due to mutations in centrosomal genes (Gabriel et al., 2016).

By studying the self-renewable and multipotent NPCs from patient-specific organoids, Gabriel et al. have revealed a surprising role of cilia in determining neural stem cell fates (Alcantara and O’Driscoll, 2014; Gabriel et al., 2016). The primary cilium is a cellular antenna that is present in almost all vertebrate cells functioning as a signaling hub. Gabriel and colleagues discovered that besides functioning as a cellular antenna, primary cilia also regulate cell cycle progression of human NPCs. In dividing cells, cilium assembly occurs during cell cycle exit (G1-G0), and disassembly coincides with cell cycle re-entry (G1-S to M). Cilium disassembly at the onset of mitosis is essential for assembling the mitotic spindle apparatus and for cell cycle re-entry. A delay or failure in cilium disassembly acts as a brake, retaining cells in G0/G1 and preventing cell cycle progression (Kim et al., 2011; Alcantara and O’Driscoll, 2014). Thus, the precise timing of cilia assembly and disassembly ensures the length of G1-S transition. These observations have defined the so-called “cilium checkpoint,” where the cilium functions as a molecular switch that regulates cell cycle progression.

Using patient-derived brain organoids, Gabriel et al. showed that NPCs harboring a mutation in CPAP has retarded cilia disassembly exhibiting an extended G1-S transition. Importantly, patient-derived brain organoids helped them to uncover that an extended G1-S transition due to a defect in cilia disassembly is sufficient to cause premature differentiation of NPCs into early neurons. Analyzing the kinetics of RGs division planes with respect to the ventricular lumen of developing brain organoids revealed that the majority of RGs of microcephaly brain organoids were vertically oriented, indicating that they tend to differentiate prematurely. This led to an overall reduction in the neural stem cell pool at the ventricular zone and, as a result thinning of the primitive cortical plate (Alcantara and O’Driscoll, 2014; Gabriel et al., 2016). Strikingly, brain organoids generated from WDR62 ablated pluripotent stem cells also led to a retarded cilium disassembly leading to decreased proliferation and premature differentiation of NPCs (Zhang et al., 2019). In summary, these works using a microcephaly brain organoid has established primary cilia as a molecular switch, regulating the homeostasis of neural epithelial tissues during brain development.

In another example of microcephaly modeling, Li et al. first cultured 3D brain organoids, which strikingly displayed in-vivo neocortex-like processes of ventricular, outer subventricular zones including laminating organization of cortical layers. Using these organoids Li and colleagues have successfully modeled the cellular defects caused by a mutation in abnormal spindle-like microcephaly-associated (ASPM) gene. It is noteworthy that the most common cause of primary human microcephaly is frequently associated with several mutations in the Aspm gene located at the MCPH5 locus. Coherent with the clinical data, patient-derived organoids displayed severe defects in structural organization displaying only a few populations of progenitor cells, which were markedly disorganized as compared to control groups. Although the cellular mechanisms for the loss of progenitor cells remain untested in this model, the smaller sized neural tissues observed in patient-derived organoids were indicative of the severely reduced brain size observed in patients.

As mentioned before, so far, the number of patient-derived organoids studied is meager. However, the mechanistic insights they offered is much more precise than derived from rodent or 2D culture models. It is also noteworthy that the underlying mechanism of NPCs depletion in patient-derived organoids is rather premature differentiation of NPCs than apparent cell death. Strikingly, most mouse models of microcephaly where the candidate genes (CDK5RAP2, CPAP, ASPM, Wdr62, and Plk4) were either completely ablated or highly overexpressed have invariably displayed apoptosis as a prominent mechanism for causing NPCs depletion and microcephaly. Unless and until cell death phenomena are prominently observed in a patient-derived organoid model, premature differentiation of NPCs leading to NPCs depletion makes most physiological sense as a mechanism causing microcephaly.

## Mechanisms Revealed by ZIKV Induced Microcephaly Modeled by Brain Organoids

It was scientific serendipity that the emergence of 3D organoid cultures has converged with the global health emergency posed by the ZIKV outbreak. Eventually, human brain organoids have pushed the frontiers of ZIKV research, as numerous studies have revealed the suitability of brain organoids in modeling microcephaly using disease-relevant ZIKV strains (Cugola et al., 2016; Qian et al., 2016; Ventura et al., 2016; Gabriel et al., 2017; Wolf et al., 2017). It is worth mentioning that several initial studies modeling ZIKV infection have used 2D cultures of NPCs and revealed that ZIKV strains are neurotropic and causing apoptosis (Tang et al., 2016). The apparent cell death phenotypes in 2D experiments did not further allow dissecting the actual cellular mechanism that caused microcephaly (Tang et al., 2016).

Subsequent studies that employed an in-depth analysis of brain organoids exposed to ZIKV at different developmental stages showed that ZIKV could directly target NPCs at the ventricular zones (Cugola et al., 2016; Dang et al., 2016; Gabriel et al., 2017; Qian et al., 2017). Detailed quantitative analysis in brain organoids revealed that ZIKV infection could cause depletion of NPCs leading to the overall size reduction of organoids as seen with genetically inherited primary microcephaly (Cugola et al., 2016; Dang et al., 2016; Gabriel et al., 2017; Qian et al., 2017). Overall, at least two different ways have been profoundly proposed to cause microcephaly phenotypes, namely, either suppression of NPC proliferation or via increased cell death. While the cell death phenotypes are apparent in infected organoids, careful interpretation is required to conclude whether the observed cell death was due to over-loading of the viral particle, increased duration of infection, cytotoxic nature of the strain, disease irrelevant strain or the combination of all.

Few works have attempted to match acquired and genetically caused microcephaly mechanisms and have proposed several causative reasons for suppressing NPC proliferation or depletion. Some of them include upregulation of toll-like receptor 3, upregulation of pro-apoptotic pathways (Dang et al., 2016), p53 activation (El Ghouzzi et al., 2018), cell cycle dysregulation (Gabriel et al., 2017), disrupting RNA-binding protein regulating NPCs growth and differentiation (Chavali et al., 2017), destabilization of adherens junction complex, and premature differentiation of NPCs. The Gargely laboratory has identified a sequence at the 3′ untranslated region of a disease-relevant ZIKV strain, which controls Musashi-1 expression post-transcriptionally. Musashi-1 is a neural RNA-binding protein that regulates the growth and differentiation of NPCs. Intriguingly, a mutation in Musashi-1 is found in primary microcephaly patients (Chavali et al., 2017). The Gargely laboratory further demonstrated that ZIKV disrupts NPCs by interfering or hijacking Musashi-1 binding to its endogenous targets (Chavali et al., 2017). By far, this is the best example showing that there is a shared mechanism between acquired and genetically caused microcephaly. An obvious question that remains unanswered is whether brain organoids derived from Musashi-1 patients exhibit microcephaly due to disrupted proliferation or differentiation equilibrium of NPCs.

While all of these studies have claimed that ZIKV could trigger premature differentiation of NPCs, Gabriel et al. have directly tested the effect of ZIKV infection in altering RGs proliferation at the ventricular zones of developing brain organoids (Gabriel et al., 2017). They showed significantly elevated numbers of RGs exhibiting vertical division planes, an indication of premature differentiation within 5 days of ZIKV infection. By performing ultrastructural analysis, the authors have also identified that there are mild structural defects in centrioles of infected RGs, a critical mechanism that could underlay the premature differentiation of RGs (Gabriel et al., 2017). In summary, these works have identified that there are indeed common mechanisms between acquired (ZIKV-induced) and genetically inherited microcephaly, which is brought to limelight by the use of human brain organoids as a test system.

## Challenges and Outlook

Even though genetic microcephaly syndromes are relatively rare, examining these disorders provide a unique advantage as they could reveal molecular mechanisms that determine NPCs maintenance, brain development, and human brain evolution in unprecedented detail. Thus, an intense effort needs to be made studying these disorders in a system that closely matches the human brain. In this scenario, the recent progress made with brain organoids strategically positions the field of microcephaly research. From experimental evidence, it has become increasingly clear that human NPCs are much more sensitive than rodents, and as a result, human NPCs are functionally impacted by mutations in the particular genes that cause microcephaly than rodent NPCs. This aspect provides an additional spotlight on the necessity for employing human brain organoids as an alternative model system to decode the most relevant mechanisms of microcephaly.

Recent work from the Kriegstein laboratory utilized large sets of comparative transcriptomes between primary human cortical cells of unknown genetic background, disease status, and brain organ (Bhaduri et al., 2020). The authors concluded that brain organoids do not recapitulate distinct cellular identities, progenitor maturation, and spatial segregation. Interestingly, their reasoning for the infidelity of organoids in this context is the activation of cellular stress pathways. While their work attempts to give a wake-up call for improving the reliability of organoids, their work did not emphasize enough of their organoid quality, since the organoids were grown for extended periods in 96 well plates in the presence of Rho-kinase (ROCK) inhibitor. Prolonged exposure to ROCK inhibitor could change the cell’s metabolism and induce the mesendodermal differentiation pathway (Maldonado et al., 2016; Le and Hasegawa, 2019). Thus, from their method, it is impossible to draw a clear boundary until which point of developmental stage organoids are accurate. It is evident that 3D brain organoids would not be able to mimic the physiological functionality of the human brain entirely but owes incredible power in revealing critical aspects of early brain development. Perhaps, their conclusion should be taken into consideration for higher-order developmental issues such as the development of complex circuitry connections in the cerebral cortex.

Comprehensive decoding of the mechanisms of microcephaly requires a repertoire of mutant models, which is the significant bottleneck at the current state of the art. Thus generating a repertoire of iPSCs from microcephaly patients will enable us to generate patient-specific 3D tissues. In our opinion, the organoid generation is less critical than acquiring stable iPSCs that harbor microcephaly mutations. This is particularly true when looking at the extraordinary progress made within the last few years in culturing 3D organoids (Gopalakrishnan, 2019). Addressing questions related to microcephaly mechanisms do not require brain organoids that are beyond the current state of the art. In other words, these questions do not necessarily depend on the need for further technological developments in the field of 3D organoid cultures. The questions of our interest mostly lie at the level of progenitor biology, and as described, several protocols have elegantly characterized the diversity of progenitors present at the ventricular zone. Introducing mutations in these cell types will allow us to dissect the molecular players and their role at the specific cell types.

An attractive alternative to patient-specific iPSCs is the genome tailoring to acquire disease-relevant patient mutations in pluripotent cells. Of note, CRISPR–Cas9-based genome editing has not been sufficiently utilized in microcephaly research using brain organoids except for a recent report, where authors have successfully eliminated the tight junction protein occludin in human embryonic stem cells. In this regard, CRISPR/Cas9-edited organoids displayed early neuronal differentiation and reduced progenitors (Bendriem et al., 2019). Remarkably, their comparative studies employing both mouse and human NPCs uncovered that human NPCs were more severely affected. Thus, applying genome tailoring in aspics to obtain organoids with patient-specific mutations will serve as a powerful tool and will allow us to generate microcephaly brain organoids to conduct a functional analysis of candidate genes in healthy human brain development.

Besides serving as a powerful *in vitro* system, organoids play a decisive role in dissecting the most likely mechanisms of microcephaly, which is NPCs depletion due to premature differentiation. Experimental evidence for this is derived from studying a few of the causative genes of primary microcephaly or ZIKV infection (Lancaster et al., 2013; Cugola et al., 2016; Gabriel et al., 2016, 2017; Dang et al., 2016; Li et al., 2017; Qian et al., 2017; Zhang et al., 2019). NPC depletion thus leads to overall brain size reduction, thinning of cortices, and impaired cortical expansion (Figure 1D). In this scenario, there are a couple of essential questions that stand out which require immediate attention. Firstly, besides standard primary microcephaly genes, several other genes also cause microcephaly, which falls under various mechanistic categories such as DNA damage, accelerated aging, mitotic delay, cytokinesis failure, transmembrane defects, cilia dysfunctions, signaling errors and autophagy (Table 1). These discoveries have pointed out that there are a wide variety of molecular and cellular mechanisms in the regulation of brain development and size determination. Do these cellular defects underlay premature NPCs differentiation? If so, during which phase of NPC proliferation, they are most prone to an attack? Studies have elucidated that retarded cilia disassembly leading to an extended G1-S transition is sufficient to trigger NPCs differentiation leading to the depletion of the symmetrically expanding NPCs pool. Thus, it remains to be tested if NPCs are vulnerable to differentiation if they are perturbed at various stages of cell cycle such as G2, and G2-M due to gene mutations that specifically target particular cell cycle stage.

As mentioned before, we are now left with the vast majority of fundamentally essential questions, which require early brain organoids displaying distinct progenitor cell layers with diverse neural precursor populations. Thus, the brain organoid systems serve as a unique platform to investigate human-specific neurodevelopmental features and hold a great promise for *in vitro* neurobiologists. In conclusion, with the emergence of 3D human brain organoids and various genomic tool kits, we are in an exciting era to dissect mechanisms of microcephaly, which will eventually help us reconstructing the complex process of the human brain development.

## Author Contributions

JG conceived the concept. EG and AR performed literature survey and helped the concept further. NA involved in conceptualizing the idea of DNA damage in microcephaly.

## Conflict of Interest

The authors declare that the research was conducted in the absence of any commercial or financial relationships that could be construed as a potential conflict of interest.
